# Democracy: the forgotten challenge for bioethics in the developing countries

**DOI:** 10.1186/1472-6939-10-3

**Published:** 2009-05-22

**Authors:** Ghaiath MA Hussein

**Affiliations:** 1 Directorate of Research, Federal Ministry of Health, Khartoum, Sudan

## Abstract

**Background:**

Bioethics as a field related to the health system and health service delivery has grown in the second half of the 20^th ^century, mainly in North America. This is attributed, the author argues, to mainly three kinds of development that took place in the developed countries at a pace different than the developing countries. They are namely: development of the health system; moral development; and political development.

**Discussion:**

This article discusses the factors that impede the development of the field of bioethics from an academic activity to a living field that is known and practiced by the people in the developing countries. They are quite many; however, the emphasis here is on role of the political structure in the developing countries and how it negatively affects the development of bioethics. It presents an argument that if bioethics is to grow within the system of health service, it should be accompanied by a parallel changes in the political mindsets in these countries.

**Summary:**

For bioethics to flourish in developing countries, it needs an atmosphere of freedom where people can practice free moral reasoning and have full potential to take their life decisions by themselves. Moreover, bioethics could be a tool for political change through the empowerment of people, especially the vulnerable.

To achieve that, the article is proposing a practical framework for facilitating the development of the field of bioethics in the developing countries.

## Background

### Bioethics and politics

Firstly, industrialized countries witnessed a huge improvement in the health technologies available for diagnosis and treatment of many diseases. This led, on one hand, to expanded expectation of the population, and, on the other hand, it expanded the mandate of the health care providers to provide better health through expanding life expectancy with better quality of life. This also led to a parallel **health system development **that was characterized by complexity of structures and functions, which made it more prone to ethical problems arising mainly from the conflicts arising from the patients' (and community in general) and the health professionals' ability to meet these expectations, provided the limitation of resources.

Secondly, there was a **moral development **as expressed in an individualistic approach to life, including issues related to health. This is best expressed by the near-consensus in the west on the importance of autonomy as the first and foremost ethical principle, among the four known ethical principles of the principalistic school of ethics formulated by Beauchamp and Childress [1].

This necessitated the presence of social and political systems that formulate clearly the rights, duties, and limits of citizenship. This has enforced the development of bioethics as a field directly related and affected by this moral structuring and development of the community.

Lastly, there was a **political development**. Most of the western countries have non-totalitarian political regimes where the political power comes from people and for the sake of people. This political system is empowered by a strong legislative and judiciary system that guards these rights, and clarifies the duties. Further, the judicial system provides legal incidents that could give guidance on many ethical issues, through its promotion of ethical criticism, moral reasoning and public engagement. Media has also grown within this political structure as a powerful tool that empowered the public engagement in ethical issues and ethical debates following them. A typical example is the case of the 41-year-old brain-injured Florida woman with persistent vegetative state, Terri Schiavo, which received 'wall-to-wall coverage in newspapers, broadcast television, cable news, the Internet, across the board'[2]. Increasing media attention led to involvement by politicians and advocacy groups, particularly those involved in the pro-life movement and disability rights, including members of the Florida Legislature, the United States Congress, and the President of the United States [3].

These factors are crucial to the development of bioethics and they are apparently absent in many developing countries. For example, my country Sudan was ruled by military totalitarian regimes for 41 years out of its 53 years of independence from the UK. It also ranked the 132^nd ^country worldwide in terms of press freedom. Other developing countries in East Asia and the Middle East have the worst press freedom records, where closing of newspapers and presenting journalists to courts because of their opinions is not uncommon in places like Egypt and Jordan [4].

Bioethics and politics inter-relate and interact and influence each other through a variety of means. First, at the theoretical level, the main 'growth factor' for bioethics is the collective moral reasoning. This needs freedom of thinking, freedom of speech, and public engagement. All of which need a political system that allow them to exist and flourish. Secondly, the practice of bioethics depends on a collective agreement on the sets of limits, rights and duties of each person in the community where it takes place. Therefore, without political development that enforces freedom of people bioethics will be no more than an academic activity.

For instance, health is managed and delivered by governments as a human right in the developed countries and they strive to achieve that through organization and allocation of services and resources. This is driven, enforced and sustained by many factors that are relatively absent in the developing countries. One main factor is the political structure that sets clearly the rules of accountability of the government to the public and their expectations, which increase in the more educated and informed populations [5]. Other factors are related to the ageing of the population where life expectancy in the developed countries almost doubles that in Sub-Saharan Africa [6]. Moreover, there are huge advances in medical sciences and technology that further increase the usually well-considered expectations of the people to better health service. Ageing populations that are well-informed and are offered the latest technology have greater expectations of what medicine, and science in general, can do for them. This is not the case in less developed countries, where death is a 'common' event, and access the basic health needs is not always achievable.

Contrarily, though WHO constitution states that 'the enjoyment the highest attainable standard of health is one of the fundamental rights of every human being'[7], there is clear discrepancy between different political regimens in delivering this 'human right'. Health is given in the Northern governments as a right-to-service, while it is, in the non-democratic countries, at least as seen by political mentality, in the developing world as a 'gift'.

This discrepancy explains why the political challenge to bioethics does not come up as a basic issue, neither in the North American literature, nor in practice. This is not the case in the developing countries where politics challenge the development and practice of bioethics.

Moreover, the clinical practice is highly affected by the political systems in many ways. The health care providers who were 'brought up' in totalitarian mono-political systems will eventually practice what have been practiced on them. This partially explains why much of our clinical practice in the developing world is characterized by 'political-like' attitudes such as paternalism, hiding or manipulating facts, and deciding on behalf of the patients. This is, one would argue, a reflection of the same attitudes adopted and practised by our politicians. They have been deciding 'what is good for us', taking crucial decisions on our behalf without involving us, and indeed tell lies and hide facts, as much as is needed to accomplish 'the highest interests of the state'.

Politics and bioethics also interact within the need of bioethics to be enforced through ethical codes, professional bodies, licensing boards, ethics committees, and a set of regulations, rules and laws. As for any profession to thrive, the public should have trust and faith in those who practice it, through its members' collective commitment to integrity and competence [8]. This is hard to achieve in non-democratic settings. As even though committees could be formed and codes could be set in place, all the bioethics enforcement factors will be overarched by freedom-restricting laws, e.g. the national security law, and other laws like those allowing 'harsh investigation techniques'-the nickname of torture, and physical punishment.

Such political setting affects bioethics enforcement structures at the levels of: 1) existence, by simply labelling ethicists as human rights' activists, which could be enough to send them to jail; 2) structure, by interfering with the process of members' selection/election; and 3) functionality, by making the codes and professional bodies overarched by the set of freedom-restricting regulations we previously mentioned, thus literally paralyzing them by depriving them from the power of taking action.

## Discussion

### Is bioethics at stake in the developing countries?

So far, I believe that bioethics in developing countries is safe from an open conflict with politicians. This safety is relative and varies from one country to another due to some factors. First, it is limited in its overall presence as an integral part of the health delivery system, which is usually left for professional regulations and bodies to run it. Second, it is confined to the level of academics and researchers who, in turn, are generally far from any real public engagement. Lastly, most of the bioethics circulated in the developing countries is 'ready-made' and the process of local reflections and adaptations, which in turn necessitates the public engagement, is not widely activated. All these factors, among others, make the politicians deal with bioethics as a 'non-threatening' academic activity. This may not be the case when the crucial exercises of engaging the public and empowering the vulnerable by giving them voices take place – and they should.

Bioethics' development and practice is relatively 'safe' from the threat of the political power used against it, as long as it is away from the touchy areas of democracy, public engagement and reflections, human rights, and accountability of health professional to the public. These concepts are barely tolerated from totalitarian politicians.

That being said, there is no bioethics without moral reasoning, which needs a clear set of rights and duties that might sooner or later provoke the politicians. This set includes for example: the limits of personal versus public rights, the person's right to be informed and to have choices, and last, but not the least, the public role in setting health policies and allocating its resources.

## Conclusion

### The way forward

In order to develop bioethics in the developing countries we have to change the way we used to practice medicine, at least in terms of dealing with our individual patients. We can not develop bioethics without involving patients in making decisions affecting their health, and indeed empowering the most vulnerable groups who are by definition in need to be empowered.

Such concepts may seem threatening to many politicians in non-democratic countries. However, there are some strategies for those working on developing bioethics in such settings that could delay, minimize, or perhaps eliminate the clash with politicians. First: moving slow but sure. Bioethics is better started in the levels least-annoying to politicians, namely the doctor-patient, and the researcher-participant relationships.

Secondly, there should be proper advocacy for bioethics as a helping tool, rather than a human right – though it is. Involvement of legislators, community representatives and religious leaders is crucial to create a public opinion positively receptive for bioethics.

Thirdly, bioethics could be introduced from a religious perspective. It is more easy to accept it when it is publicly known that most of the universal religions are now developing their own set of bioethics, e.g. Catholic, Jehovah Witnesses, Buddhist, Jewish, Islamic bioethics, etc. as a response to the secular morality in which bioethics grew in the West [9]. Care is needed not to present bioethics as contradicting to religion, which (usually mixed with local cultures) plays a key role in affecting the political agendas. This influence is lesser in the secular West, where religious bioethics is one of the approaches and not the main approach.

Lastly, great care is needed to not to present bioethics as a "western product". This might be difficult, when for instance, as in Sudan, all those who had degrees in ethics were trained in the US and Canada. However, it is possible with proper adaptation of literature, adequate integration of local values, especially religion, to build national bioethics centres that work on both developing local literature and offer training in bioethics. West-African Bioethics Society and Agha Khan University Bioethics Center are good examples.

Although some work was conducted to assess the situation of research ethics in developing countries, including Sudan [10-12], these authors overlooked the issues related to the effect of the political context on their structure and function. This is a good area for research and development of bioethics practice to be not only sensitive to inequalities attributed to gender, geography, and tribe, but also sensitive to public representation and active engagement.

In conclusion, bioethics should be utilized as a powerful tool to increase the choices of people in areas of the world where the right of free choice is absent, or restricted by non-democratic political regimes.

## Competing interests

The author declares that they have no competing interests.

## Authors' contributions

GH has developed the argument, reviewed the literature and prepared the whole article's manuscript for submission.

## Author's information

The author is a graduate of the MHSc. Program in Bioethics of the Joint Centre for Bioethics at the University of Toronto, Canada. He is now the deputy director of the research directorate at the Federal Ministry of Health, Sudan. He is an instructor and curriculum advisor on bioethics for some Sudanese universities.

## Pre-publication history

The pre-publication history for this paper can be accessed here:


